# Crystal structure and Hirshfeld surface analysis of (3*Z*)-7-meth­oxy-3-(2-phenyl­hydrazinyl­idene)-1-benzo­furan-2(3*H*)-one

**DOI:** 10.1107/S2056989021007891

**Published:** 2021-08-06

**Authors:** Zeliha Atioğlu, Mehmet Akkurt, Ulviyya F. Askerova, Sevinc H. Mukhtarova, Rizvan K. Askerov, Sixberth Mlowe

**Affiliations:** a Department of Aircraft Electrics and Electronics, School of Applied Sciences, Cappadocia University, Mustafapaşa, 50420 Ürgüp, Nevşehir, Turkey; b Department of Physics, Faculty of Sciences, Erciyes University, 38039 Kayseri, Turkey; c Organic Chemistry Department, Baku State University, Z. Xalilov str. 23, Az, 1148 Baku, Azerbaijan; d University of Dar es Salaam, Dar es Salaam University College of Education, Department of Chemistry, PO Box 2329, Dar es Salaam, Tanzania

**Keywords:** crystal structure, 2,3-di­hydro-1-benzo­furan ring system, dimers, hydrogen bonds, Hirshfeld surface analysis

## Abstract

In the title compound, C_15_H_12_N_2_O_3_, pairs of mol­ecules are linked into dimers by N—H⋯O hydrogen bonds, forming an *R* ^2^_2_(12) ring motif, with the dimers stacked along the *a* axis. These dimers are connected through π–π stacking inter­actions between the centroids of the benzene and furan rings of their 2,3-di­hydro-1-benzo­furan ring systems. Furthermore, there exists a C—H⋯π inter­action that consolidates the crystal packing. A Hirshfeld surface analysis indicates that the most important contacts are H⋯H (40.7%), O⋯H/H⋯O (24.7%), C⋯H/H⋯C (16.1%) and C⋯C (8.8%).

## Abstract 2

Pairs of mol­ecules in the crystal are linked into dimers by N—H⋯O hydrogen bonds, forming an 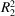
(12) ring motif. The dimers are connected through π–π stacking inter­actions between the centroids of the benzene and furan rings of their 2,3-di­hydro-1-benzo­furan ring systems. C—H⋯π inter­actions consolidate the crystal packing.

## Chemical context   

Hydrazones are a versatile class of organic ligands that have extensive applications in synthetic transformations, the synthesis of bioactive compounds, the design of materials and in coordination chemistry (Ma *et al.*, 2017*a* ▸,*b* ▸; Viswanathan *et al.*, 2019 ▸). Moreover, metal complexes of hydrazone ligands have been successfully applied as catalysts in organic synthesis (Gurbanov *et al.*, 2018 ▸). The properties of metal-hydrazonates can be regulated by the design of ligands through the involvement of non-covalent-bond donor or acceptor substituents (Ma *et al.*, 2020 ▸, 2021 ▸; Mahmudov *et al.*, 2013 ▸). Supra­molecular networks of all dimensions in the crystal structures of hydrazone compounds or metal-hydrazonates, resulting from extensive hydrogen-bonding and other types of inter­molecular inter­actions, have been reported (Gurbanov *et al.*, 2020*a* ▸; Kopylovich *et al.*, 2011 ▸). Thus, the attachment of suitable substituents or synthons to hydrazone ligands can improve their functional properties and the catalytic or biological activity of the corresponding coordination compounds (Mizar *et al.*, 2012 ▸; Gurbanov *et al.*, 2020*a* ▸,*b* ▸; Khalilov *et al.*, 2018*a* ▸,*b* ▸; Maharramov *et al.*, 2018 ▸; Shihkaliyev *et al.*, 2019 ▸; Shixaliyev *et al.*, 2014 ▸).
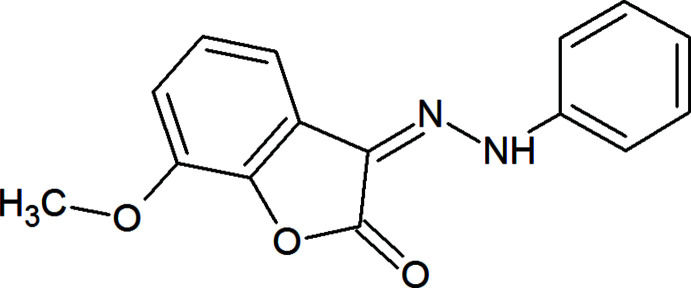


In a continuation of our work in this context (Atioğlu *et al.*, 2020 ▸, 2021 ▸), we have synthesized a new hydrazone compound, (3*Z*)-7-meth­oxy-3-(2-phenyl­hydrazinyl­idene)-1-benzo­furan-2(3*H*)-one, which shows multiple inter­molecular non-covalent inter­actions.

## Structural commentary   

**Figure 1:**
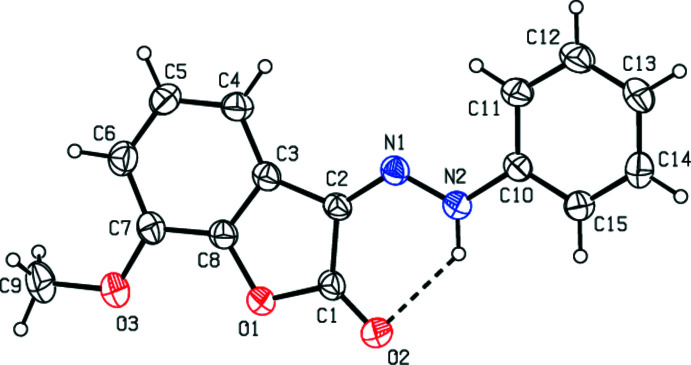
The title mol­ecule with the labelling scheme and displacement ellipsoids drawn at the 30% probability level. The intra­molecular N—H⋯O hydrogen bond is shown as a dashed line.

In the title compound, the mol­ecular conformation is stabilized by an intra­molecular N2—H1⋯O2 hydrogen bond, forming an *S*(6) ring motif (Table 1 ▸, Fig. 1 ▸; Bernstein *et al.*, 1995 ▸). The 2,3-di­hydro-1-benzo­furan ring system (O1/C1–C8) is essentially planar [maximum deviation of 0.016 (2) Å for O1] and subtends a dihedral angle of 5.32 (14)° with the phenyl ring (C10–C15).

## Supra­molecular features   

**Figure 2:**
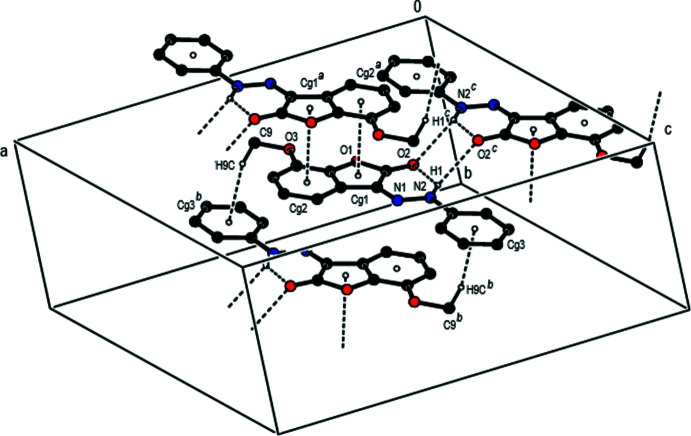
Inter­molecular N—H⋯O hydrogen bonds, C—H⋯π inter­actions and π–π stacking inter­actions (shown as dashed lines) in the title compound. [Symmetry codes: (*a*) 1 − *x*, −*y*, 1 − *z*; (*b*) 1 − *x*, 1 − *y*, 1 − *z*; (*c*) 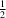 − *x*, 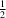 − *y*, 1 − *z*].

In the crystal, pairs of mol­ecules are linked into dimers by inter­molecular N—H⋯O hydrogen bonds, forming an 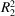
(12) ring motif (Table 1 ▸). These dimers are stacked along the *a* axis and connected by π–π stacking inter­actions between the centroids of the benzene and furan rings of their 2,3-di­hydro-1-benzo­furan ring systems [*Cg*1⋯*Cg*2(1 − *x*, − *y*, 1 − *z*) = 3.5316 (19) Å, slippage = 0.352 Å, where *Cg*1 and *Cg*2 are the centroids of the benzene (C3–C8) and furan (O1/C1–C3/C8) rings, respectively] (Figs. 2 ▸, 3 ▸ and 4 ▸). Furthermore, there exists a C—H⋯π inter­action between the H9*C* atom of the methyl group C9 and the centroid of the phenyl ring (C10–C15).

## Hirshfeld surface analysis   

**Figure 5:**
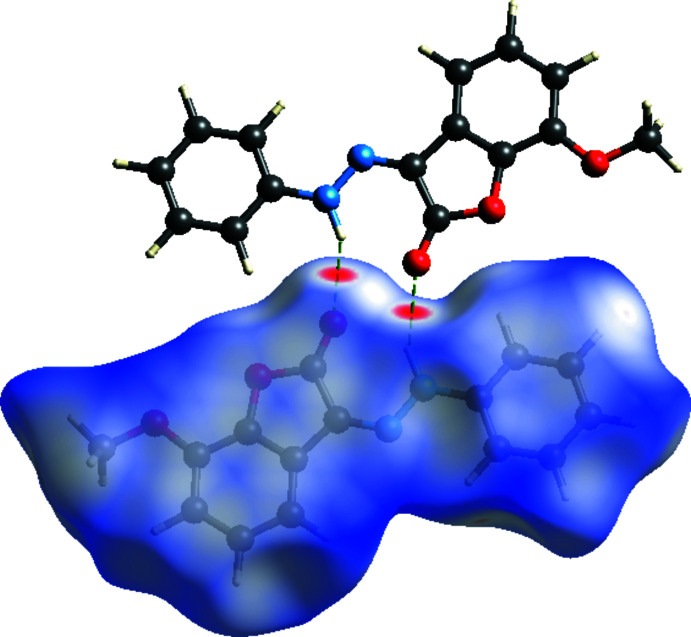
View of the three-dimensional Hirshfeld surface of the title compound plotted over *d*_norm_ in the range −0.1718 to 1.3843 a.u. The two N—H⋯O hydrogen bonds forming the dimer are depicted as dashed lines.

**Figure 6:**
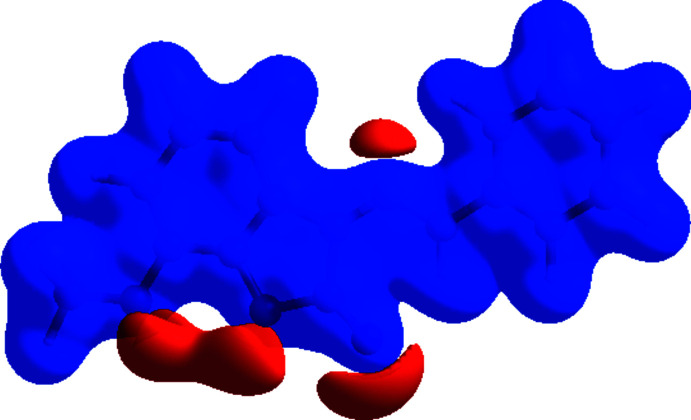
View of the three-dimensional Hirshfeld surface of the title compound plotted over electrostatic potential energy in the range −0.0500 to 0.0500 a.u. using the STO-3 G basis set at the Hartree–Fock level of theory. The hydrogen-bond donors and acceptors are viewed as blue and red regions, respectively, around atoms, corresponding to positive and negative potentials.

*Crystal Explorer 17.5* (Turner *et al.*, 2017 ▸) was used to calculate the Hirshfeld surfaces and generate the two-dimensional fingerprint plots. Hirshfeld surfaces allow for the display of inter­molecular inter­actions by using distinct colours and intensities to indicate short and long contacts, as well as the relative strength of the inter­actions. The three-dimensional Hirshfeld surface of the title compound plotted over *d*_norm_ in the range −0.1718 to 1.3843 a.u. is shown in Fig. 5 ▸. The N2—H1⋯O2 inter­actions, which play a key role in the mol­ecular packing of the title compound, are responsible for the red spot that occurs around O2. The bright-red spots appearing near O2 and hydrogen atom H1 indicate their roles as donors and/or acceptors in hydrogen-bonding; they also appear as blue and red regions corresponding to positive and negative potentials on the Hirshfeld surface mapped over electrostatic potential (Spackman *et al.*, 2008 ▸) shown in Fig. 6 ▸. Here the blue regions indicate positive electrostatic potential (hydrogen-bond donors), while the red regions indicate negative electrostatic potential (hydrogen-bond acceptors).

**Figure 7:**
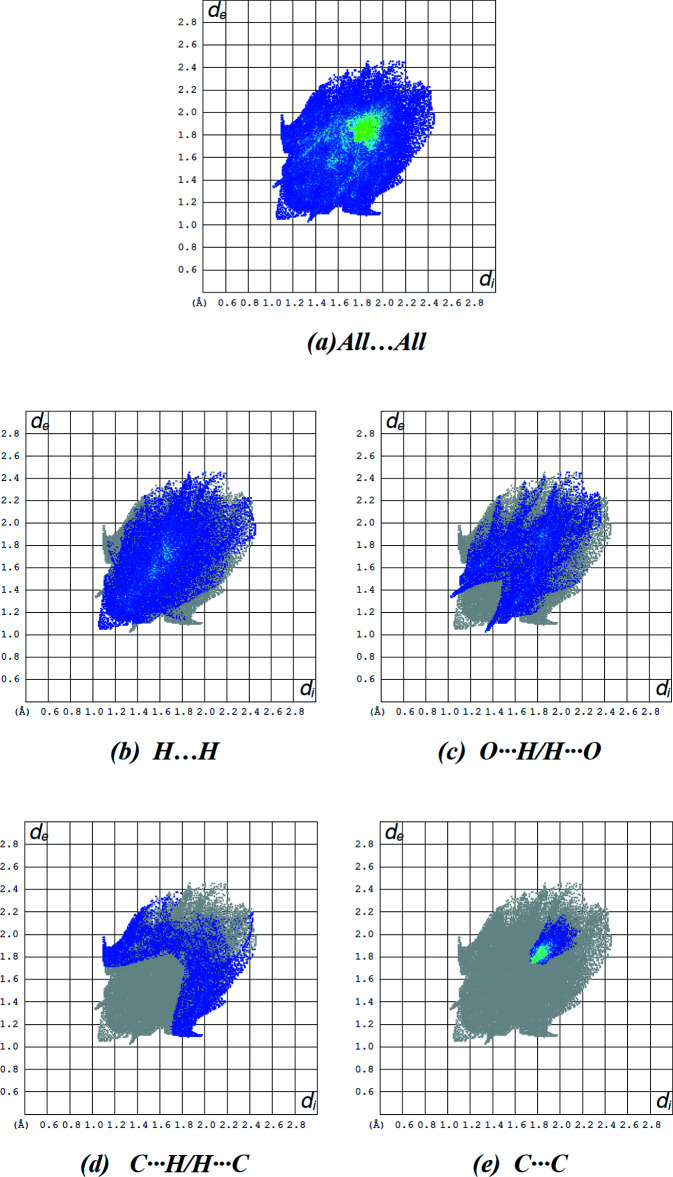
The full two-dimensional fingerprint plots for the title compound, showing (*a*) all inter­actions, and delineated into (*b*) H⋯H, (*c*) O⋯H/H⋯O, (*d*) C⋯H/H⋯C and (*e*) C⋯C inter­actions. The *d*_i_ and *d*_e_ values are the closest inter­nal and external distances (in Å) from given points on the Hirshfeld surface contacts.

The overall two-dimensional fingerprint plot for the title compound is given in Fig. 7 ▸ *a*, and those delineated into H⋯H, O⋯H/H⋯O, C⋯H/H⋯C and C⋯C contacts are shown in Fig. 7 ▸ *b*–*e*, while numerical details of the different contacts are given in Table 2 ▸. The percentage contributions to the Hirshfeld surfaces from the various inter­atomic contacts are as follows: H⋯H (Fig. 7 ▸ *b*; 40.7%), O⋯H/H⋯O (Fig. 7 ▸ *c*; 24.7%), C⋯H/H⋯C (Fig. 7 ▸ *d*; 16.1%) and C⋯C (Fig. 7 ▸ *e*; 8.8%). Other minor contributions to the Hirshfeld surface are from N⋯C/C⋯N (3.8%), N⋯H/H⋯N (3.5%), O⋯C/C⋯O (1.9%), O⋯N/N⋯O (0.4%) and O⋯O (0.2%) contacts.

## Database survey   

A search of the Cambridge Crystallographic Database (CSD version 5.40, update of September 2019; Groom *et al.*, 2016 ▸) gave 763 hits for structures with a hydrazone moiety. Five structures that are closely related to the title compound are: 2-(4-nitro-1*H*-imidazol-1-yl)-*N*′-[1-(pyridin-2-yl)ethyl­idene]acetohydrazide (TODMEH; Oliveira *et al.*, 2019 ▸); 2-(2-nitro-1*H*-imidazol-1-yl)-*N*′-[1-(pyridin-2-yl)ethyl­idene]aceto­hy­dra­zide (TODMIL; Oliveira *et al.*, 2019 ▸); 2-(4-nitro-1*H*-imidazol-1-yl)-*N*′-[phen­yl(pyridin-2-yl)methyl­idene]acetohydrazide (TODMOR; Oliveira *et al.*, 2019 ▸); 2-(4-nitro-1*H*-imidazol-1-yl)-*N*′-[phen­yl(pyridin-2-yl)methyl­idene]acetohydrazide (TODMUX; Oliveira *et al.*, 2019 ▸) and 1,1′-[1,3-phenyl­enebis(2,2-di­chloro­ethene-1,1-di­yl)]bis­(phenyl­diazene) (EXIWOA; Shikhaliyev *et al.*, 2021 ▸).

TODMEH and TODMOR crystallize in the monoclinic space group *P*2_1_/*c* with *Z* = 4. TODMIL crystallizes in the monoclinic space group *I*2/*a* with *Z* = 8 and TODMUX crystallizes in the triclinic space group *P* 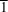 with *Z* = 2. EXIWOA crystallizes in the monoclinic space group *P*2_1_/c with *Z* = 4. The *E* conformation in TODMEH, TODMIL and TODMUX is stabilized by a strong inter­molecular N—H⋯O inter­action. These inter­actions lead to the formation of dimeric structural arrangements. In the crystal packing of TODMOR, an inter­molecular N—H⋯N inter­action results in a zigzag structural arrangement, with the formation of chains along the crystallographic *b* axis. Non-classical inter­molecular C—H⋯N and C—H⋯O inter­actions are also observed in the crystal structures of TODMEH, TODMIL, TODMOR and TODMUX. In EXIWOA, mol­ecules are linked by C—H⋯π, C—Cl⋯π, Cl⋯Cl and Cl⋯H inter­actions, forming a three-dimensional supra­molecular network.

## Synthesis and crystallization   

A 20 ml screw-neck vial was charged with dimethyl sulfoxide (DMSO; 10 ml), (*E*)-2-{[2-(3,5-di­methyl­phen­yl)hydrazineyl­idene]meth­yl}phenol (240 mg, 1 mmol), tetra­methyl­ethyl-enedi­amine (TMEDA; 295 mg, 2.5 mmol), CuCl (2 mg, 0.02 mmol) and CCl_4_ (20 mmol, 10 equiv). After 1–3 h (until TLC analysis showed complete consumption of the corres­ponding Schiff base), the reaction mixture was poured into a 0.01 *M* solution of HCl (100 mL, pH = 2-3), and extracted with di­chloro­methane (3 × 20 ml). The combined organic phase was washed with water (3 × 50 ml), brine (30 ml), dried over anhydrous Na_2_SO_4_ and concentrated *in vacuo* in a rotary evaporator. The residue was purified by column chromatography on silica gel using appropriate mixtures of hexane and di­chloro­methane (*v*/*v* = 3/1–1/1). Colourless solid (yield 65%); m.p. 475 K. Analysis calculated for C_15_H_12_N_2_O_3_ (*M* = 268.27): C 67.16, H 4.51, N 10.44; found: C 67.11, H 4.47, N 10.35%. ^1^H NMR (300 MHz, CDCl_3_) δ 12.13 (*s*, 1H, NH), 6.91–7.43 (8H, Ar), 3.99 (*s*, 3H, OCH_3_). ^13^C NMR (75 MHz,CDCl_3_) δ 186.20, 161.87, 150.65, 141.76, 129.60, 125.09, 124.44, 123.87, 114.90, 112.74, 111.44, 108.76, 56.46. ESI–MS: *m*/*z*: 269.26 [*M* + H]^+^. Crystals suitable for X-ray analysis were obtained by slow evaporation of a di­chloro­methane solution.

## Refinement details   

Crystal data, data collection and structure refinement details are summarized in Table 3 ▸. The H atom of the NH group was located in a difference-Fourier map and refined freely [N2—H1 = 0.92 (4) Å]. H atoms bonded to C atoms were positioned geometrically and refined using a riding model, with C—H = 0.93 or 0.96 Å, and with *U*_iso_(H) = 1.2*U*_eq_(C) for aromatic or 1.5*U*_eq_(C) for methyl H atoms. Owing to poor agreement between observed and calculated intensities, seven outliers, (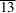 7 1), (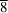 6 13), (13 7 0), (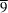 5 19), (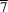 5 20), (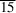 5 12) and (0 6 16), were omitted in the final cycles of refinement.

## Supplementary Material

Crystal structure: contains datablock(s) I. DOI: 10.1107/S2056989021007891/wm5614sup1.cif

Structure factors: contains datablock(s) I. DOI: 10.1107/S2056989021007891/wm5614Isup2.hkl

Supporting information file. DOI: 10.1107/S2056989021007891/wm5614Isup3.cml

CCDC reference: 1984938

Additional supporting information:  crystallographic information; 3D view; checkCIF report

## Figures and Tables

**Figure 3:**
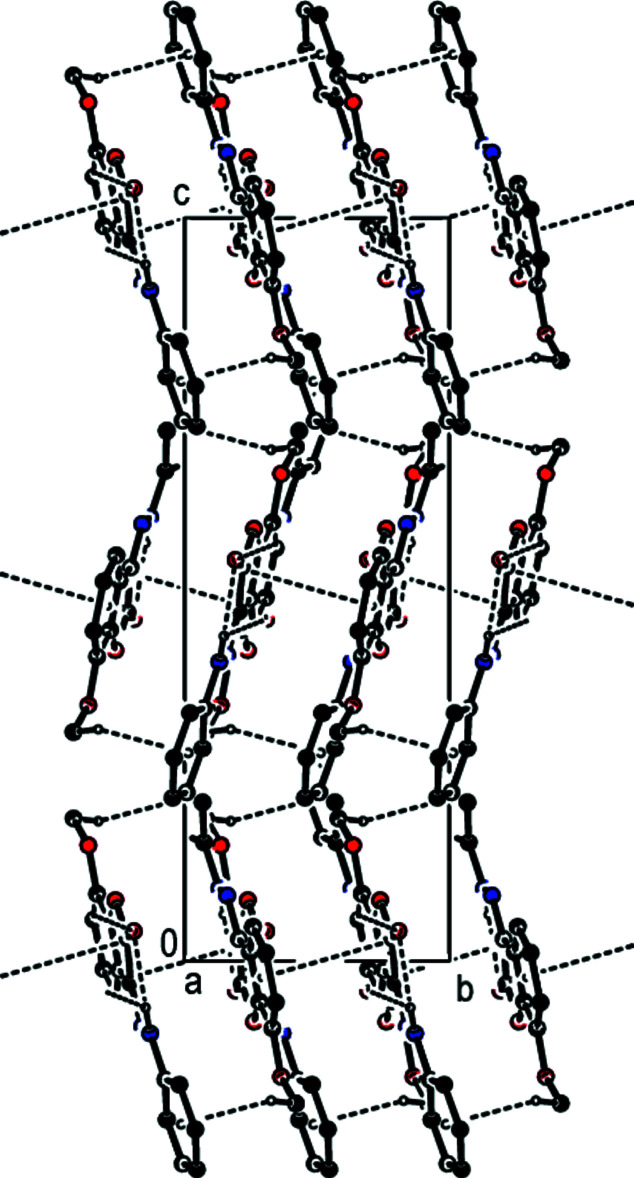
A view of the mol­ecular packing of the title compound along the *a-*axis direction. Inter­molecular inter­actions are depicted as in Fig. 2 ▸.

**Figure 4:**
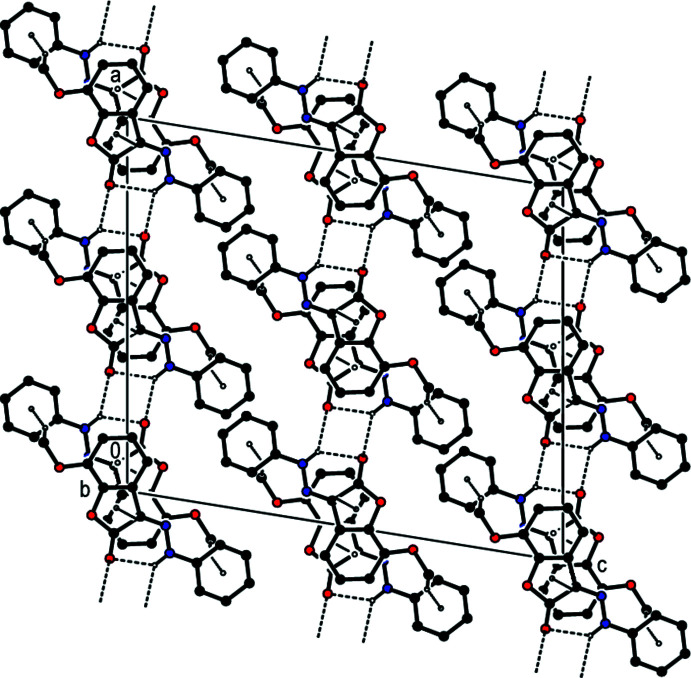
A view of the mol­ecular packing of the title compound along the *b-*axis direction. Inter­molecular inter­actions are depicted as in Fig. 2 ▸.

**Table 1:** Hydrogen-bond geometry (Å, °) *Cg*3 is the centroid of the C10–C15 phenyl ring.

*D*—H⋯*A*	*D*—H	H⋯*A*	*D*⋯*A*	*D*—H⋯*A*
N2—H1⋯O2	0.92 (4)	2.14 (3)	2.843 (3)	133 (3)
N2—H1⋯O2^i^	0.92 (4)	2.44 (4)	3.181 (4)	138 (3)
C9—H9*C*⋯*Cg*3^ii^	0.96	2.70	3.555 (4)	149

Symmetry codes: (i) -x+{\script{1\over 2}}, -y+{\script{1\over 2}}, -z+1; (ii) 1-x, 1-y, 1-z.

**Table 2:** Inter­atomic contacts of the title compound (Å)

Contact	Distance	Symmetry operation
H1⋯O2	2.44	{1\over 2} − *x*, {1\over 2} − *y*, 1 − *z*
H9*B*⋯N2	2.91	1 − *x*, −*y*, 1 − *z*
H9*C*⋯C11	2.93	1 − *x*, 1 − *y*, 1 − *z*
H5*A*⋯H15*A*	2.51	{1\over 2} + *x*, −{1\over 2} + *y*, *z*
C9⋯H14*A*	2.85	{1\over 2} + *x*, {1\over 2} − *y*, −{1\over 2} + *z*
H11*A*⋯H11*A*	2.31	1 − *x*, *y*, {3\over 2} − *z*
C15⋯H13*A*	3.07	{1\over 2} − *x*, −{1\over 2} + *y*, {3\over 2} − *z*

**Table 3:** Experimental details

Crystal data	
Chemical formula	C_15_H_12_N_2_O_3_
*M*_r_	268.27
Crystal system, space group	Monoclinic, *C*2/*c*
Temperature (K)	296
*a*, *b*, *c* (Å)	17.436 (2), 7.2485 (7), 20.595 (2)
β (°)	99.181 (4)
*V* (Å^3^)	2569.6 (5)
*Z*	8
Radiation type	Mo *K*α
μ (mm^−1^)	0.10
Crystal size (mm)	0.49 × 0.15 × 0.06

Data collection	
Diffractometer	Bruker APEXII CCD
Absorption correction	Multi-scan (*SADABS*; Krause *et al.*, 2015 ▸)
*T*_min_, *T*_max_	0.629, 0.745
No. of measured, independent and observed [*I* > 2σ(*I*)] reflections	12800, 2427, 1463
*R*_int_	0.085
(sin θ/λ)_max_ (Å^−1^)	0.616

Refinement	
*R*[*F*^2^ > 2σ(*F* ^2^)], *wR*(*F* ^2^), *S*	0.073, 0.152, 1.01
No. of reflections	2427
No. of parameters	187
H-atom treatment	H atoms treated by a mixture of independent and constrained refinement
Δρ_max_, Δρ_min_ (e Å^−3^)	0.18, −0.18

Computer programs: *APEX3* and *SAINT* (Bruker, 2017 ▸), *SHELXS* (Sheldrick, 2008 ▸), *SHELXL* (Sheldrick, 2015 ▸), *ORTEP-3 for Windows* (Farrugia, 2012 ▸) and *PLATON* (Spek, 2020 ▸).

## supplementary crystallographic information

### Crystal data

**Table:**

C_15_H_12_N_2_O_3_	*F*(000) = 1120
*M**_r_* = 268.27	*D*_x_ = 1.387 Mg m^−^^3^
Monoclinic, *C*2/*c*	Mo *K*α radiation, λ = 0.71073 Å
*a* = 17.436 (2) Å	Cell parameters from 2240 reflections
*b* = 7.2485 (7) Å	θ = 2.4–26.3°
*c* = 20.595 (2) Å	µ = 0.10 mm^−^^1^
β = 99.181 (4)°	*T* = 296 K
*V* = 2569.6 (5) Å^3^	Prism, colourless
*Z* = 8	0.49 × 0.15 × 0.06 mm

### Data collection

**Table:**

Bruker APEXII CCD diffractometer	1463 reflections with *I* > 2σ(*I*)
φ and ω scans	*R*_int_ = 0.085
Absorption correction: multi-scan (*SADABS*; Krause *et al.*, 2015)	θ_max_ = 26.0°, θ_min_ = 2.0°
*T*_min_ = 0.629, *T*_max_ = 0.745	*h* = −21→21
12800 measured reflections	*k* = −8→8
2427 independent reflections	*l* = −25→25

### Refinement

**Table:**

Refinement on *F*^2^	Secondary atom site location: difference Fourier map
Least-squares matrix: full	Hydrogen site location: mixed
*R*[*F*^2^ > 2σ(*F*^2^)] = 0.073	H atoms treated by a mixture of independent and constrained refinement
*wR*(*F*^2^) = 0.152	*w* = 1/[σ^2^(*F*_o_^2^) + (0.0517*P*)^2^ + 2.9831*P*] where *P* = (*F*_o_^2^ + 2*F*_c_^2^)/3
*S* = 1.01	(Δ/σ)_max_ < 0.001
2427 reflections	Δρ_max_ = 0.18 e Å^−^^3^
187 parameters	Δρ_min_ = −0.18 e Å^−^^3^
0 restraints	Extinction correction: SHELXL2016/6 (Sheldrick 2015), Fc^*^=kFc[1+0.001xFc^2^λ^3^/sin(2θ)]^-1/4^
Primary atom site location: difference Fourier map	Extinction coefficient: 0.0015 (4)

### Special details

**Table:**

Geometry. All esds (except the esd in the dihedral angle between two l.s. planes) are estimated using the full covariance matrix. The cell esds are taken into account individually in the estimation of esds in distances, angles and torsion angles; correlations between esds in cell parameters are only used when they are defined by crystal symmetry. An approximate (isotropic) treatment of cell esds is used for estimating esds involving l.s. planes.

### Fractional atomic coordinates and isotropic or equivalent isotropic displacement parameters (Å^2^)

**Table:**

	*x*	*y*	*z*	*U*_iso_*/*U*_eq_	
O1	0.42001 (12)	0.2382 (3)	0.41831 (10)	0.0546 (6)	
O2	0.31175 (13)	0.3077 (4)	0.45997 (10)	0.0648 (7)	
O3	0.53382 (14)	0.1359 (4)	0.34426 (11)	0.0728 (8)	
N1	0.42389 (15)	0.3396 (4)	0.58811 (12)	0.0477 (7)	
N2	0.35184 (16)	0.3699 (4)	0.59750 (13)	0.0500 (7)	
C1	0.38107 (19)	0.2841 (5)	0.46932 (14)	0.0483 (8)	
C2	0.43742 (17)	0.2955 (4)	0.52959 (13)	0.0434 (8)	
C3	0.51208 (17)	0.2536 (4)	0.51198 (14)	0.0450 (8)	
C4	0.58718 (18)	0.2419 (5)	0.54600 (16)	0.0591 (10)	
H4A	0.598100	0.265759	0.590895	0.071*	
C5	0.64493 (19)	0.1937 (5)	0.51096 (18)	0.0653 (10)	
H5A	0.695811	0.185900	0.532762	0.078*	
C6	0.6297 (2)	0.1564 (5)	0.44427 (17)	0.0615 (10)	
H6A	0.670362	0.122386	0.422508	0.074*	
C7	0.55574 (19)	0.1688 (5)	0.40953 (15)	0.0521 (9)	
C8	0.49847 (17)	0.2183 (4)	0.44538 (14)	0.0466 (8)	
C9	0.5936 (2)	0.0804 (6)	0.30818 (17)	0.0778 (12)	
H9A	0.571774	0.063916	0.262729	0.117*	
H9B	0.615969	−0.033713	0.325700	0.117*	
H9C	0.633113	0.173711	0.311913	0.117*	
C10	0.33773 (18)	0.4270 (4)	0.65966 (14)	0.0467 (8)	
C11	0.3957 (2)	0.4326 (5)	0.71354 (14)	0.0594 (10)	
H11A	0.445790	0.395598	0.709579	0.071*	
C12	0.3796 (2)	0.4926 (5)	0.77285 (16)	0.0671 (11)	
H12A	0.419310	0.497293	0.808808	0.081*	
C13	0.3062 (2)	0.5458 (5)	0.78029 (16)	0.0640 (10)	
H13A	0.295701	0.585028	0.820967	0.077*	
C14	0.2482 (2)	0.5402 (5)	0.72659 (16)	0.0604 (10)	
H14A	0.198194	0.577300	0.730814	0.073*	
C15	0.26373 (19)	0.4799 (5)	0.66640 (15)	0.0538 (9)	
H15A	0.224071	0.475136	0.630425	0.065*	
H1	0.313 (2)	0.352 (5)	0.5627 (17)	0.082 (13)*	

### Atomic displacement parameters (Å^2^)

**Table:**

	*U*^11^	*U*^22^	*U*^33^	*U*^12^	*U*^13^	*U*^23^
O1	0.0484 (13)	0.0750 (18)	0.0406 (11)	−0.0036 (12)	0.0072 (10)	−0.0031 (11)
O2	0.0484 (15)	0.092 (2)	0.0533 (14)	−0.0061 (13)	0.0049 (11)	−0.0059 (12)
O3	0.0659 (16)	0.103 (2)	0.0527 (14)	0.0041 (14)	0.0200 (12)	−0.0074 (14)
N1	0.0488 (16)	0.0505 (18)	0.0441 (14)	−0.0068 (13)	0.0082 (12)	0.0010 (12)
N2	0.0471 (17)	0.060 (2)	0.0427 (15)	−0.0017 (14)	0.0062 (13)	−0.0044 (13)
C1	0.049 (2)	0.054 (2)	0.0428 (17)	−0.0112 (17)	0.0118 (15)	0.0010 (15)
C2	0.0476 (18)	0.044 (2)	0.0375 (16)	−0.0077 (15)	0.0050 (14)	0.0020 (14)
C3	0.0498 (19)	0.042 (2)	0.0431 (16)	−0.0085 (15)	0.0082 (14)	0.0052 (14)
C4	0.053 (2)	0.073 (3)	0.0489 (18)	−0.0091 (18)	0.0014 (16)	0.0038 (17)
C5	0.044 (2)	0.083 (3)	0.068 (2)	−0.0021 (18)	0.0070 (18)	0.012 (2)
C6	0.054 (2)	0.067 (3)	0.067 (2)	−0.0014 (18)	0.0194 (18)	0.0100 (19)
C7	0.058 (2)	0.051 (2)	0.0499 (19)	−0.0049 (17)	0.0159 (17)	0.0025 (16)
C8	0.0481 (19)	0.047 (2)	0.0447 (17)	−0.0073 (15)	0.0070 (15)	0.0063 (15)
C9	0.092 (3)	0.084 (3)	0.066 (2)	0.011 (2)	0.038 (2)	−0.006 (2)
C10	0.052 (2)	0.047 (2)	0.0404 (16)	−0.0068 (15)	0.0080 (15)	−0.0010 (15)
C11	0.055 (2)	0.076 (3)	0.0456 (18)	0.0031 (18)	0.0016 (16)	−0.0101 (17)
C12	0.074 (3)	0.080 (3)	0.0446 (19)	0.003 (2)	0.0019 (18)	−0.0106 (18)
C13	0.078 (3)	0.069 (3)	0.047 (2)	−0.002 (2)	0.0174 (19)	−0.0081 (17)
C14	0.060 (2)	0.061 (3)	0.065 (2)	0.0041 (18)	0.0248 (19)	0.0021 (18)
C15	0.050 (2)	0.060 (2)	0.0511 (19)	−0.0028 (17)	0.0061 (16)	0.0037 (16)

### Geometric parameters (Å, º)

**Table:**

O1—C1	1.380 (3)	C6—C7	1.375 (5)
O1—C8	1.400 (3)	C6—H6A	0.9300
O2—C1	1.205 (3)	C7—C8	1.381 (4)
O3—C7	1.359 (4)	C9—H9A	0.9600
O3—C9	1.431 (4)	C9—H9B	0.9600
N1—C2	1.304 (3)	C9—H9C	0.9600
N1—N2	1.320 (3)	C10—C15	1.374 (4)
N2—C10	1.404 (4)	C10—C11	1.377 (4)
N2—H1	0.92 (4)	C11—C12	1.367 (4)
C1—C2	1.457 (4)	C11—H11A	0.9300
C2—C3	1.438 (4)	C12—C13	1.369 (5)
C3—C8	1.378 (4)	C12—H12A	0.9300
C3—C4	1.386 (4)	C13—C14	1.375 (5)
C4—C5	1.374 (4)	C13—H13A	0.9300
C4—H4A	0.9300	C14—C15	1.381 (4)
C5—C6	1.383 (5)	C14—H14A	0.9300
C5—H5A	0.9300	C15—H15A	0.9300
			
C1—O1—C8	106.9 (2)	C3—C8—C7	123.8 (3)
C7—O3—C9	116.7 (3)	C3—C8—O1	112.3 (3)
C2—N1—N2	119.5 (3)	C7—C8—O1	123.9 (3)
N1—N2—C10	119.5 (3)	O3—C9—H9A	109.5
N1—N2—H1	118 (2)	O3—C9—H9B	109.5
C10—N2—H1	123 (2)	H9A—C9—H9B	109.5
O2—C1—O1	121.0 (3)	O3—C9—H9C	109.5
O2—C1—C2	130.6 (3)	H9A—C9—H9C	109.5
O1—C1—C2	108.4 (3)	H9B—C9—H9C	109.5
N1—C2—C3	126.1 (3)	C15—C10—C11	119.4 (3)
N1—C2—C1	127.2 (3)	C15—C10—N2	118.6 (3)
C3—C2—C1	106.7 (2)	C11—C10—N2	122.1 (3)
C8—C3—C4	119.4 (3)	C12—C11—C10	120.1 (3)
C8—C3—C2	105.7 (3)	C12—C11—H11A	120.0
C4—C3—C2	134.9 (3)	C10—C11—H11A	120.0
C5—C4—C3	117.6 (3)	C11—C12—C13	121.2 (3)
C5—C4—H4A	121.2	C11—C12—H12A	119.4
C3—C4—H4A	121.2	C13—C12—H12A	119.4
C4—C5—C6	122.1 (3)	C12—C13—C14	118.8 (3)
C4—C5—H5A	119.0	C12—C13—H13A	120.6
C6—C5—H5A	119.0	C14—C13—H13A	120.6
C7—C6—C5	121.3 (3)	C13—C14—C15	120.5 (3)
C7—C6—H6A	119.4	C13—C14—H14A	119.8
C5—C6—H6A	119.4	C15—C14—H14A	119.8
O3—C7—C6	126.6 (3)	C10—C15—C14	120.0 (3)
O3—C7—C8	117.5 (3)	C10—C15—H15A	120.0
C6—C7—C8	115.9 (3)	C14—C15—H15A	120.0
			
C2—N1—N2—C10	−176.6 (3)	C4—C3—C8—C7	0.8 (5)
C8—O1—C1—O2	−179.2 (3)	C2—C3—C8—C7	−178.7 (3)
C8—O1—C1—C2	0.8 (3)	C4—C3—C8—O1	−179.3 (3)
N2—N1—C2—C3	−178.2 (3)	C2—C3—C8—O1	1.2 (4)
N2—N1—C2—C1	3.7 (5)	O3—C7—C8—C3	179.2 (3)
O2—C1—C2—N1	−1.8 (6)	C6—C7—C8—C3	−0.3 (5)
O1—C1—C2—N1	178.3 (3)	O3—C7—C8—O1	−0.6 (5)
O2—C1—C2—C3	179.9 (4)	C6—C7—C8—O1	179.8 (3)
O1—C1—C2—C3	−0.1 (3)	C1—O1—C8—C3	−1.3 (3)
N1—C2—C3—C8	−179.0 (3)	C1—O1—C8—C7	178.6 (3)
C1—C2—C3—C8	−0.7 (3)	N1—N2—C10—C15	172.0 (3)
N1—C2—C3—C4	1.5 (6)	N1—N2—C10—C11	−7.6 (5)
C1—C2—C3—C4	179.9 (4)	C15—C10—C11—C12	−0.8 (5)
C8—C3—C4—C5	−0.4 (5)	N2—C10—C11—C12	178.7 (3)
C2—C3—C4—C5	178.9 (4)	C10—C11—C12—C13	0.8 (6)
C3—C4—C5—C6	−0.5 (6)	C11—C12—C13—C14	−0.7 (6)
C4—C5—C6—C7	1.0 (6)	C12—C13—C14—C15	0.7 (5)
C9—O3—C7—C6	0.9 (5)	C11—C10—C15—C14	0.8 (5)
C9—O3—C7—C8	−178.5 (3)	N2—C10—C15—C14	−178.8 (3)
C5—C6—C7—O3	179.9 (3)	C13—C14—C15—C10	−0.8 (5)
C5—C6—C7—C8	−0.6 (5)		

### Hydrogen-bond geometry (Å, º)

*Cg*3 is the centroid of the C10–C15 phenyl ring.

**Table:**

*D*—H···*A*	*D*—H	H···*A*	*D*···*A*	*D*—H···*A*
N2—H1···O2	0.92 (4)	2.14 (3)	2.843 (3)	133 (3)
N2—H1···O2^i^	0.92 (4)	2.44 (4)	3.181 (4)	138 (3)
C9—H9*C*···*Cg*3^ii^	0.96	2.70	3.555 (4)	149

Symmetry codes: (i) −*x*+1/2, −*y*+1/2, −*z*+1; (ii) −*x*+1, −*y*+1, −*z*+1.

### Percentage contributions of interatomic contacts to the Hirshfeld surface for the title compound

**Table:**

Contact	Percentage contribution
H···H	40.7
O···H/H···O	24.7
C···H/H···C	16.1
C···C	8.8
N···C/C···N	3.8
N···H/H···N	3.5
O···C/C···O	1.9
O···N/N···O	0.4
O···O	0.2
